# Chromatin Modification and Remodeling in Heart Development

**DOI:** 10.1100/tsw.2006.315

**Published:** 2006-04-24

**Authors:** Paul Delgado-Olguín, Jun K. Takeuchi, Benoit G. Bruneau

**Affiliations:** ^1^ Programmes in Cardiovascular Research and Developmental Biology, The Hospital for Sick Children, Toronto, ON, Canada; ^2^ The Heart and Stroke/Richard Lewar Centre of Excellence at the University of Toronto, Toronto, ON, M5S 1A8, Canada; ^3^ Department of Molecular & Medical Genetics, University of Toronto, Toronto, ON, M5S 1A8, Canada

**Keywords:** heart, chromatin, histones, SWI/SNF, embryogenesis, gene expression, transcription factors

## Abstract

In organogenesis, cell types are specified from determined precursors as morphogenetic patterning takes place. These events are largely controlled by tissue-specific transcription factors. These transcription factors must function within the context of chromatin to activate or repress target genes. Recent evidence suggests that chromatin-remodeling and -modifying factors may have tissue-specific function. Here we review the potential roles for chromatin-remodeling and -modifying proteins in the development of the mammalian heart.

During heart development, many transcription factors play key roles in regulating differentiation and patterning of the heart[1,2]. Furthermore, these transcription factors often interact together to activate or repress downstream target genes, resulting in fine combinatorial regulation of complex morphogenetic and regulatory patterns. However, it is not known how these interactions are regulated in higher-order chromatin, in which they must function to active their target genes. It is also unknown how chromatin-modifying or -remodeling factors control the function of gene regulatory elements in specific tissues or cell types, such as cardiac myocytes.

## CHROMATIN-REMODELING FACTORS IN HEART DEVELOPMENT

In the nucleus, DNA is wrapped around histones, which are packed into nucleosomes, which are further condensed into chromatin[3,4]. The structure of chromatin is plastic, with some “closed” states keeping gene activation repressed, and in others the chromatin is “open”, allowing fluid access to transcription factors and the transcriptional machinery. Remodeling or repositioning of nucleosomes is key to transition between these two states. To accommodate this, nature has developed complexes that remodel chromatin by ATP-dependent unraveling of DNA from nucleosomes.

Several ATP-dependent chromatin-remodeling complexes have been identified in eukaryotes and these are exemplified by the yeast Swi/Snf complex (originally identified in yeast as "mating type switching and sucrose nonfermenting" mutants) and its related complexes[5]. In mammals, the Swi/Snf complex is represented by several related polymorphic complexes, referred to as the Brg1/Brm-associated factor (BAF) complexes[6,7]. The BAF complexes are large (2 MDa) and contain one of two ATPases, and at least 11 variable subunits, which are highly conserved from yeast to humans. The mammalian BAF complex is illustrated in Fig. 1A. The two BAF complex ATPases are Brg1 (Brahma-related gene 1) and Brm, the name of which derives from *Brahma* of the *Drosophila* Swi2/Snf2 homolog[8]. After remodeling the nucleosomes by function of chromatin-remodeling factors, it is generally thought that BAF complexes are important for transcriptional activation by unwinding nucleosomes and allowing RNA polymerase II to access transcriptional initiations sites[5]. Furthermore, Brg1 can directly bind specific transcription factors, such as Gata factors[9]. Indeed, Brg1 binding to promoter elements is one of the first and most critical steps in transcription at several loci[10]. It is not clear, however, whether there are there any specific roles for BAF complexes in organogenesis.

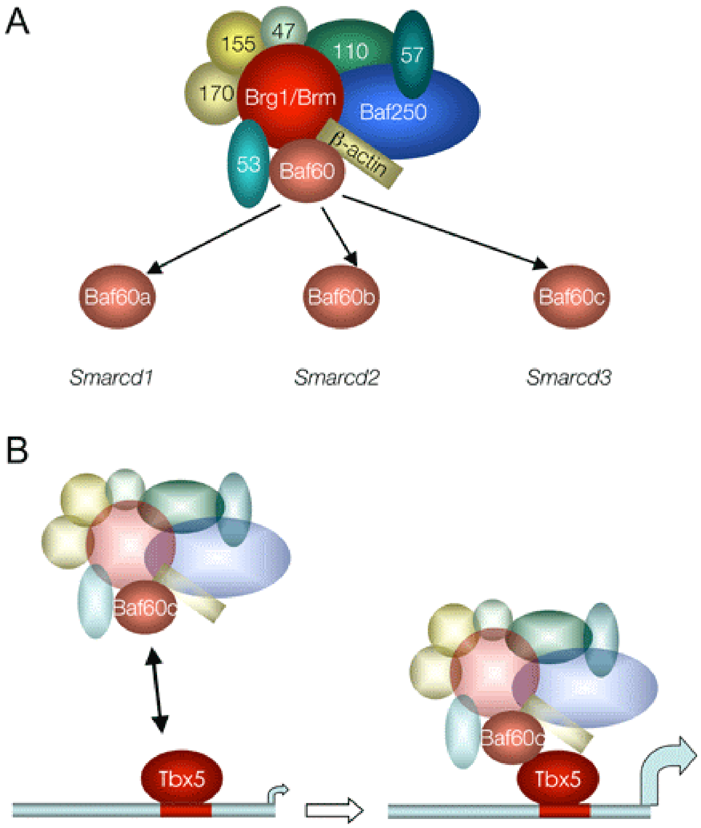

**FIGURE 1.** (A) The BAF complex. Baf60a–c are shown as examples of polymorphic subunits, with their gene name (*Smarcd1–3*); (B) model for recruitment of the BAF complex by Tbx5 via interactions with Baf60c.

## Chromatin-Remodeling Complexes: Insight from Mouse Knockouts

Studies of other BAF complex subunits in mice have provided little insight into the roles played by ATP-dependent chromatin-remodeling complexes in postimplantation embryonic development. Knockout mice of several BAF complex subunits such as Brg1, Baf155, and Snf5 demonstrated that the BAF complex is necessary for embryonic development from very early stages, as mice lacking either one of these subunits do not develop past the implantation stage[11,12,13,14]. The zebrafish *young* mutant, which is a deletion of *Brg1*, has defective differentiation of the retina cell, but has undefined defects in heart formation[15,16]. The loss of Brm results in live-born and almost completely normal mice that have slight defects in cell proliferation[17]. During organogenesis, Brg1 may be the primary effector of BAF complex function, but it is clear that in some tissues, Brg1 and Brm function cooperatively[18]. In the immune system, one of the specific roles of BAF complexes in lineage switching has been uncovered by using dominant-negative Baf57 or T-cell specific deletion of *Brg1*[19,20,21]. This result indicates BAF complexes may participate in the specification of tissue-specific cell fate in organogenesis. Snf2h and Snf2l, which are core ATPases of ISWI (Imitation Switch) type of SWI/SNF chromatin-remodeling factors, are expressed in the developing heart. The loss of Snf2h is also required for early mouse development as Snf2h-/- embryos die during the peri-implantation stage[22].

## Baf60c and Heart Development

Baf60c is one isoform of three 60-kDa BAF complex subunits that are conserved in the yeast and fly Swi/Snf complexes (Fig. 1A). Baf60a, Baf60b, and Baf60c are encoded by three distinct genes, *Smarcd1*, *Smarcd2*, and *Smarcd3*. Baf60a has been characterized as important for linking the glucocorticoid receptor (GR) to BAF complex[23] and has also been shown to be important for c-fos/c-jun transcriptional activity[24]. On the other hand, no information exists regarding Baf60b function. We have shown that Baf60a is not expressed until e9.5 in mouse development, and that Baf60b exhibits specific expression in the developing gut and limb buds[25]. Baf60c is expressed very early in the precardiac mesoderm at late gastrulation and prenodal plate stages, and at later stages retains tissue-specific expression in the heart, as well as somites and the developing central nervous system[25,26]. This expression of Baf60c suggested that it could act as a tissue-specific subunit of the BAF complex, perhaps serving to remodel chromatin at specific loci during heart development.

This novel concept in tissue-specific transcriptional regulation, in which a tissue-specific-expressing, chromatin-remodeling complex is critical for specific aspects of organogenesis, was shown by loss of function experiments that demonstrated dramatic and specific defects in heart and somite formation in mouse embryo. To address the *in vivo* function of Baf60c in mouse embryo, we employed *in vivo* short hairpin RNA interference (shRNAi)[27]. Embryos with severe knockdown of Baf60c had malformed hearts, along with decreased expression of the "working myocardium" marker *ANF* (*Nppa*), loss of the trabecular markers *Bmp10* and *Irx3*, and loss of several outflow tract marker genes (*Fgf10*, *Pitx2*, and *Bmp4*). Normal expression of *Gata4*, *Nkx2-5*, and *Tbx5*, which are key transcription factors in heart development, was seen at 9.0–9.5, but it was interesting that at least one of their target genes, *ANF*, had severely decreased expression levels, suggesting that Baf60c might have a role in tissue-specific regulation via interactions with specific transcriptional factors in heart development. Indeed, coimmunoprecipitation experiments showed that Baf60c could act as a bridge, creating or reinforcing molecular interactions between cardiac transcription factors (Gata4, Nkx2-5, and Tbx5) and Brg1, thus bringing the BAF complex to target genes bound by Gata4, Nkx2-5, or Tbx5 (Fig. 1B). We finally concluded that Baf60c conferred tissue specificity to the BAF complex during heart development.

## Baf180 and Heart Development

Another insight into roles of chromatin-remodeling complexes during heart development was the result of Polybromo protein Baf180 knockout mice[28]. Baf180 is part of the BAF-related PBAF complex, which is able to potentiate transcriptional activation mediated by nuclear receptors, such as RXRa, VDR, and PPARg. Loss of Baf180 does not lead to early embryonic lethality, but instead uncovers a specific role for the PBAF complex in late aspects of cardiac chamber maturation, which caused very thin cardiac wall and fewer trabeculations and these heart phenotypes of Baf180 null embryos revealed a similar to that of RXRa knockout mice[29]. These results indicate that the PBAF complex may have critical roles in embryogenesis, especially the late stage of heart formation, and have different roles from Baf60c during cardiac development. Thus, diversity of transcriptional regulation by two related, but distinct, chromatin-remodeling complexes, BAF and PBAF, may regulate distinct pathways in heart development.

## HISTONE MODIFICATIONS AND CARDIAC DEVELOPMENT

Histones are covalently modified by acetylation[30], methylation[31], phosphorylation[32], ubiquitination[33], sumoylation[34], and ribosylation[35], resulting in changes in chromatin structure and thereby affecting transcriptional regulation[36,37]. Fig. 2 illustrates the general concepts of active and repressive chromatin modifications. As for many cellular contexts, there is evidence that suggests the relevance of histone modifications in the regulation of cardiac development. In recent years, histone acetylation has been at the center of attention since histone acetyltransferases (HATs) and histone deacetylases (HDACs) have been demonstrated to be important regulators of aspects of cardiac gene expression, with implications in the control of specific physiologic and developmental responses[38]. Moreover, histone acetylation has recently been proposed as a potential target for therapeutic approaches in controlling cardiac hypertrophy[39,40]. However, histone methylation, despite being known for more than 40 years[41], has recently emerged as an important part of a code, in which post-translational chromatin modifications are indispensable for the establishment of orchestrated developmental gene expression patterns[42,43]. It is reasonable to assume then that a developmental role for histone modifications in organogenesis may exist and be highly regulated; what this code is during heart development remains to be uncovered.

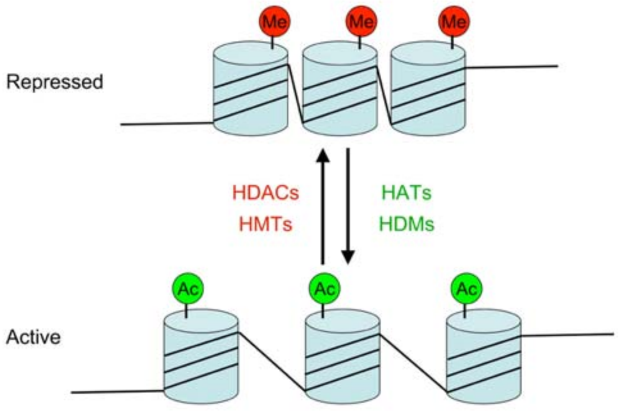

**FIGURE 2**. Transitions from repressed to active chromatin and vice versa are regulated by a balance of active chromatin marks (e.g., histone H3-K9 acetylation, shown as green circles) and repressive marks (e.g., histone H3-K27 methylation, shown as red circles). Enzymes that add or remove these marks control the balance between active and repressed chromatin. For example, histone acetyltransferases (HATs) and histone demethlyases (HDMs) generally function to promote active chromatin, while histone deacetylases (HDACs) and histone methyltransferases (HMTs) promote inactive chromatin.

### Establishment of Histone Modifications

HATs and HDACs mediate histone acetylation and deacetylation, generally associated with transcriptional activation and repression, respectively[30]. The addition of mono-, di-, or trimethyl groups at the e-N group of lysine and arginine residues of histones H3, H4, and H1b is catalyzed by histone methyltransferases (HMTs), through the SET domain[31,37,44]. In general, methylation of H3-K4, 17, and 36 is associated with transcriptionally active regions, whereas methylation marks at H3-K9 and 27 are found mainly at inactive chromatin[31,37]. The presence of such marks affects the accessibility of transcription factors and/or constitutes docking sites for proteins that spread a regulatory signal. For example, heterochromatin protein 1 (HP1) recognizes di- or trimethylated H3-K9 through a protein domain called the chromodomain[45,46], targeting SUV39H1 that methylates H3 in K9 of the next nucleosome, creating new HP1 recognition sites, thereby contributing to the establishment of long-term transcriptional repression[47]. The addition of methyl marks seems to be affected by the presence of other histone modifications like phosphorylation and ubiquitination. Phosphorylation of H3-S10 and acetylation of H3-K14 correlate with the dissociation of HP1 from methylated H3-K9[48]. Ubiquitination of H2B-K123 is required for the further methylation of H3-K4 and H3-K9, as demonstrated in yeast[49]. Moreover, methylation of H3-K9 prevents further acetylation by p300[50]. The presence of histone modification in such a combinatorial fashion predicts the requirement of highly versatile “reading machines” that recognize and sense and/or establish a particular chromatin state, and perform a specific response leading to a modulated expression pattern. The bromo- and chromodomain-containing proteins have so far been identified as the adaptors between histone acetylation and methylation marks, respectively, and a regulatory response. In agreement with this idea, the composition of polycomb repressor complexes changes during cellular differentiation, which affect their histone substrate specificities *in vitro*[51]. This scenario could imply that the protein complexes including bromo- or chromodomain-containing factors could need specific transcription factors to be targeted to regulatory regions. In *Drosophila*, the polycomb repressor complex 1 (PRC1), which mediates HDAC and HMT activities[52,53], is recruited to DNA at polycomb response elements, where it presumably methylates H3-K9 through EZh2, leading to recruitment of HP1. No polycomb response elements have been identified in mammals, opening the possibility of the involvement of tissue-specific transcription factors by a homologous mechanism, as shown for Ezh2, and HDACs, which are recruited by muscle and cardiac-specific transcription factors to gene regulatory regions (see below).

## Transcription Factors and the Establishment of Histone Modifications during Cardiac Development

Several transcriptional regulators important for heart development have been shown to rely on histone-modifying enzymes. The SET and MYND domain protein mBop, which is essential for cardiac development and is expressed in cardiac and skeletal muscle precursors, had been demonstrated to act as a transcriptional regulator of *Hand2*, through an HDAC activity-dependent mechanism[54]. mBop also interacts with Irx5 to recruit HDACs, thus conferring negative regulatory potential to Irx5 in establishing potassium channel gradients in the heart[55]. Recently it has been proposed that Bop may act as a methyltransferase[56], suggesting that this protein may be a versatile muscle-restricted histone-modifying protein.

Serum response factor (SRF) plays a central role in the regulation of cardiac development, presumably through modulating the function of Nkx2.5, GATA4, and myocardin[57,58,59]. Mice with a cardiac-specific deletion of SRF die due to abnormal myocardium, dilated cardiac chambers, poor trabeculation, and a disorganized interventricular septum[60]. SRF establishes a functional interaction with HDAC4, which represses SRF-dependent transactivation that, in turn, is relieved by the activation of Ca2+/calmodulin-dependent protein kinase, which leads to nuclear export of HDAC4[61]. These observations suggest that histone deacetylation could participate in the global regulation of cardiogenesis. In agreement with this idea, the expression SRF seems to be dependent on interactions between T-box transcription factors such as Tbx2 and Tbx5, and the HAT activity of the MYST family HAT TIP60[62].

In addition, the homeodomain protein Hop interacts with HDAC2 in a protein complex that is presumably recruited to a SRF-dependent promoter[63]. Under this scenario, it seems that SRF activity is intimately linked to HDAC activity.

The potential role of histone acetylation in cardiac development is highlighted by the fact that p300 null mice present cardiac defects like enlarged heart cavity, severe pericardial effusion, and reduced ventricular trabeculation, as well as impaired expression of cardiac genes[64]. p300 was further demonstrated to interact with cardiac transcription factors like MEF2D, which interacts with Emb, a class VI POU domain protein, and regulates the expression of the cardiac actin gene through binding a distal enhancer[65]. CBP/p300 interacts with Cited2, a TFAP2 coactivator[66], which is essential for cardiac development, as shown through a murine knockout approach. The resulting mice present abnormal conditions like atrial and ventricular septal defects, overriding aorta, double-outlet right ventricle, persistent truncus arteriosus, and right-sided aortic arches[67]. p300 also acts as a transcriptional coactivator of the GATA family of transcription factors, which have critical functions in the regulation of cardiac development[68,69], arguing in favor of the idea that histone acetylation globally affects cardiac gene expression.

Tbx5, mutations in which cause cardiac and limb defects characteristic of Holt-Oram Syndrome, is a T-box transcription factor critical for early cardiac morphogenesis and gene expression[70,71]. Tbx5 has recently been found to establish functional interaction with TAZ, a WW-domain-containing transcriptional regulator, which recruits the HATs p300 and PCAF, enhancing the Tbx5-dependent transactivation of the *Nppa* promoter[72]. Interestingly, such a cooperative effect is precluded by *TBX5* mutations found in Holt-Oram Syndrome patients, opening the possibility that Tbx5-mediated involvement of histone acetylation patterns is a key mechanism for congenital heart defects observed in Holt-Oram Syndrome.

The evidence presented so far supports the notion of the involvement of transcription factors in the recruitment of chromatin modifiers to cardiac gene regulatory regions during heart development. It is noteworthy that despite the demonstration of the existence of functional interactions between histone-modifying enzymes and cardiac transcription factors, to date, the precise modifications induced by these enzymes over specific target promoters, and thereby their possible implications in the regulation of the cardiac transcriptional program, have been overlooked.

## A Role of Histone Methylation in Cardiac Development?

A careful study of histone methylation function during mammalian development has been precluded since mice deficient for HMTs Suv39h, G9a, and Ezh2 are not viable, and die at early peri-implantation stages[73,74,75]. Studies employing tissue-specific *Ezh2* knockout mice led to the association of this HMT with B-cell development[76], which in turn opened the possibility of the involvement of Ezh2 and thereby histone methylation in other developmental processes. Interestingly, the involvement of other polycomb-group genes in cardiac development had been demonstrated. Mice lacking the Polycomb family gene *Rae*28 have several cardiac abnormalities including defects in atrioventricular canal formation and incomplete looping[77].

Histone methylation marks are established early during development, and during cardiogenesis, differentiation genes are expressed in response to signaling cascades, favoring the existence of two nonexclusive possible scenarios: (1) temporal-spatial expression of positive regulators determine the activation of gene expression and (2) negative epigenetic regulatory marks are lost during this process. The first scenario has been the subject of extensive study, which has led to the clear identification of cardiac transcription factors and their functional interactions[1]. However, the second scenario is poorly understood, particularly in the matter of histone methylation. In this regard, histone methylation of H3 on K9 and K27, which are marks associated with transcriptional repression, could play a role in keeping the expression of late differentiation markers off during early differentiation, which could imply Ezh2 in such global negative regulatory effect. This hypothesis could be sustained by the fact that *Ezh2* is progressively repressed during other processes like B-cell differentiation[76] and myogenesis[78], in which *Ezh2* down-regulation correlates with the expression of myogenin. Moreover, activation in muscle cells of the expression of MHCIIb and MCK genes correlate with loss of Ezh2, HDACI, and YYI, the presence of which correlates with H3-K27 di- and trimethylation, as well as recruitment of SRF and MyoD to the corresponding promoters. YYI, which recognizes the CarG-box, is thought to recruit Ezh2 to the MHCIIb and MCK promoters[78], raising the possibility that histone methylation could be mediated by Ezh2 through interactions with general or specific transcription factors. However, a homologous mechanism regulating cardiac muscle gene expression has not been described. Interestingly, the regulatory regions of several cardiac genes like *Mlc2*, *B-type natriuretic peptide*, and *cardiac alpha-actin* respond to YYI[79,80,81], opening the possibility that negative histone methylation marks could contribute to the establishment of their expression patterns, thereby affecting cardiogenesis. Moreover, YY1 interacts with the polycomb protein Edd[82], which is indispensable for the HMT activity of the PRC1[83]. Interestingly, it has been recently demonstrated that at E10.5, monomethylation of H4-K20 is underrepresented or absent in myosin heavy-chain positive cells of cardiac muscle, whereas trimethylation of this residue is enriched, as shown by immunofluorescence[84]. This dynamic pattern in the distribution of methylation marks strengthens the possibility that histone methylation could play a role in the control of cardiac development. On the other hand, external stimuli can induce rearrangements in the distribution of epigenetic marks. In agreement with this idea, induction of shear stress over mouse embryonic stem cells enhance lysine acetylation of histone H3-K14, serine phosphorylation of H3-S10, and methylation of H3-K79, as well as early induction of cardiovascular markers including smooth muscle actin, smooth muscle protein 22-alpha, platelet-endothelial cell adhesion molecule-1, VEGF receptor 2, myocyte enhancer factor-2C (MEF2C), and alpha-sarcomeric actin[85].

Despite the evidence suggesting the involvement of polycomb-group genes and histone methylation in the regulation of cardiac development, the function of HMTs in the embryonic heart remains largely unknown, leaving a deep gap in our knowledge of the epigenetic regulation of cardiac development. Some interesting candidate genes have recently been identified. As mentioned above, Bop may act as a HMT[56]. Indeed, Bop acts as a transcriptional repressor of cardiac gene expression. Also, the recent discovery of the JmjC domain as a signature-conserved HDM motif implies that Jumonji, a member of the JmjC-containing family of proteins, could be a cardiac HDM[86,87,88]. Jumonji acts primarily as a repressor and requires the JmjC domain to accomplish this, implying that Jumonji's activity as a HDM is important for transcriptional repression.

Our present knowledge indicates that heart development is controlled by cardiac transcription factors, which temporal-spatial expression and combinatorial interactions lead to the regulated expression of cardiac genes[1,2]. Such transcription factors expression depends on signaling cascades modulated by BMP, Notch, and Wnts, establishing a hierarchical regulatory scenario; however, the histone code hypothesis adds another level of gene control, in which early establishment of chromatin epigenetic signals could dictate the expression of signaling intermediaries, early cardiac transcription factors or both.

## FUTURE DIRECTIONS

The elucidation of the histone methylation marks pattern during cardiac development would constitute a first approach to contribute to our understanding of the potential function of histone methylation in cardiogenesis. Another important issue could be constituted by the identification of HMT gene targets. Additionally, the potential role of cardiac transcription factors in the establishment of histone methylation marks via interactions with polycomb repressor complexes may indicate that the regulation of cardiac development, and tissue-specific gene expression as a whole, is far more complex than previously thought. Combined with an understanding of the genetic program that requires tissue-specific subunits of the ATP-dependent chromatin-remodeling complexes such as Baf60c, the regulation of cardiac morphogenesis by chromatin modification and remodeling may reveal a few interesting twists.
